# Characterization of a novel polysaccharide from *red ginseng* and its ameliorative effect on oxidative stress injury in myocardial ischemia

**DOI:** 10.1186/s13020-022-00669-6

**Published:** 2022-09-24

**Authors:** Yuanpei Lian, Maomao Zhu, Bing Yang, Xianfeng Wang, Jingqi Zeng, Yanjun Yang, Shuchen Guo, Xiaobin Jia, Liang Feng

**Affiliations:** 1 School of Traditional Chinese Pharmacy, China Pharmaceutical University, Nanjing, 211198 People’s Republic of China; 2 Changzhou Affiliated Hospital of Nanjing University of Chinese Medicine, Changzhou, People’s Republic of China 213003

**Keywords:** Red ginseng polysaccharides, Structure characterization, Acute myocardial ischemia, Nrf2 pathway

## Abstract

**Background:**

Red ginseng (RG) was widely used as traditional Chinese medicine (TCM) or dietary supplement. However, few researches had been reported on the red ginseng polysaccharide (RGP).

**Methods:**

In this study, a novel heteropolysaccharide named RGP1-1 was fractionated sequentially by DEAE-52 column and Sephadex G-100 gel column. The primary structure of RGP1-1, including glycosyl linkages, molecular weight, monosaccharide composition, morphology and physicochemical property were conducted by nuclear magnetic resonance (NMR), gas chromatography-mass spectrometer (GC–MS), atomic force microscope (AFM), scanning electron microscope (SEM), differential scanning calorimetry-thermogravimetric analysis (DSC-TG) and so on. The effect of RGP1-1 in preventing and treating myocardial ischemia was evaluated by an animal model isoprenaline (ISO) induced mice.

**Results:**

RGP1-1, with a homogeneous molecular weight of 5655 Da, was composed of Glc and Gal in the ratio of 94.26:4.92. The methylation and NMR analysis indicated the backbone was composed of → 1)-Glcp-(4 → and → 1)-Galp-(4 →, branched partially at O-4 with α-D-Glc*p-*(1 → residue. Morphology and physicochemical property analysis revealed a triple-helical conformation, flaky and irregular spherical structure with molecule aggregations and stable thermal properties of RGP1-1. And it contained 6.82 mV *zeta* potential, 117.4 nm partical size and polymerization phenomenon. Furthermore, RGP1-1 possessed strong antioxidant activity in vitro and in vivo, RGP1-1 could decrease cardiomyocyte apoptosis and myocardium fibrosis of mice in histopathology and it could decrease significantly the serum levels of cardiac troponin (cTnI), aspartate aminotransferase (AST), lactate dehydrogenase (LDH), malondialdehyde (MDA). Western blot analysis showed that RGP1-1 can increase the expression of main protein Nuclear factor E2-related factor 2(Nrf2), NAD(P)H:quinone oxidoreductase 1 (NQO1), heme oxygenase-1(HO-1) and kelch-like ECH-associated protein1(keap1) in oxidative stress injure progress, and therefore regulate the pathway of Nrf2/HO-1.

**Conclusion:**

The above findings indicated that RGP1-1 had an improving effect on ISO-induced myocardial ischemia injury in mice, as novel natural antioxidant and heart-protecting drugs.

**Supplementary Information:**

The online version contains supplementary material available at 10.1186/s13020-022-00669-6.

## Introduction

Various natural polysaccharides had aroused great interests for researchers because of their anti-oxidant and anti-cardiovascular disease activities [1–4]. At present, a variety of polysaccharides with good protection and treatment for cardiovascular diseases (CVD) had been isolated from natural resource [5–7]. CVD include coronary heart disease, angina pectoris and atherosclerosis, especially myocardial ischemia (MI). Therefore, natural polysaccharides have great potential for the prevention and treatment of MI as a pharmaceutical and functional food ingredient.

Red ginseng (RG) was obtained from fresh ginseng processed by simple or continuous steaming/drying. RG was widely used as traditional Chinese medicine (TCM) or dietary supplement in east Asian countries such as China, South Korea and Japan for thousand years [8, 9]. Compared with fresh ginseng or white ginseng, the processed RG sustained a higher biological activity and fewer side effects [10]. The chemical composition will change during processing of ginseng to RG. The species and relative contents of ginsenosides in RG were more than those of fresh ginseng or white ginseng [11], and ginsenosides were converted into moderately polar oligosaccharide saponins which can significantly improve its absorption and enhance its bioavailability [12, 13]. Besides, as ginsenosides was hydrolyzed into glycosides, the sugars, oligosaccharides and reducing sugars in RG had increased to varying degrees [14]. In addition, Maillard reaction occurs during steaming to generate Maillard products, which helps scavenge free radicals [15]. The neurotoxic component of dencichin in ginseng was significantly reduced after high temperature processing, thereby reducing the toxic and side effects [16]. Some studies had shown that RG could improve and protect MI injury [17, 18]. Lim et al. [18] found that Korean RG could reduce the oxidative stress response to improve myocardial ischemia and play a role in protecting the heart.

In recent years, most of studies were focused on the biological activities and other practical applications of ginsenoside in RG [19, 20]. However, few researches had been reported on the red ginseng polysaccharide (RGP). In the process of fresh ginseng processing to RG, the saccharide in ginseng were hydrolyzed to varying degrees under the influence of conditions such as heat, enzymes and acids. Moreover, the sugar content of white ginseng was higher than that of red ginseng due to the loss of water [21]. It reported that the polysaccharide content of fresh ginseng was reduced from 51.32 to 33.18% after processing to RG [14]. Interestingly, the antioxidant activity of RGP was significantly higher than that of ginseng polysaccharides [22]. It is worth nothing that RGP is also one of the main components with a variety of active functions, such as antitumor, immunomodulatory, antioxidant, and free radical scavenging activities. Currently, there is no application of RGP in the treatment of MI injury caused by oxidative stress. In addition, the activity of polysaccharide was closely related to its structure, but the structure composition of RGP were unclear, and it limited the development and utilization of this precious resource.

Therefore, based on the protective effect of RG and other polysaccharides against MI, we will hypothesize that polysaccharides from RG may have preventive and therapeutic effects on MI. A novel polysaccharide named RGP1-1 was purified and isolated from RG, and the possible mechanism for its activity to protective effect of MI was clarified. In addition, the activity of polysaccharides was closely related to its structure, the primary structure of RGP1-1, including glycosyl linkages, molecular weight, monosaccharide composition, morphology and physicochemical property were conducted by NMR, GC–MS, AFM, SEM, DSC-TG and so on. The present study establishes the basis for further understanding RGP and provides necessary reference for the use of RGP1-1 as a functional food and potential alternative medicine to protect MI injury.

## Materials and methods

### Drugs and reagents

*Red ginseng* (Lot. 20200626) was obtained from Anhui Hetian Chinese Medicine Co., Ltd. (Bozhou, China) and identified by Long Wang (China Pharmaceutical University). d-glucose, d-galactose, d-galacturonic acid, l-rhamnose, d-glucuronic acid, d-mannose, l-arabinose d-Fructose, d-Xylose and dextran were purchased from Sigma Chemical Co., Ltd (St. Louis, MO, USA). DEAE-52 and Sephadex-G100 were purchased from GE Healthcare Life Sciences (Piscataway, NJ, USA). Isopropyl hydrochloride adrenaline (ISO) was provided by Sigma Chemical Co., Ltd (St. Louis, MO, USA). Chloral hydrate was purchased from Sinopharm Shanghai Chemical Reagent Co., Ltd (Shanghai China). Creatine kinase (CK), lactate dehydrogenase (LDH), aspartate transaminase (AST), glutathione peroxidase (GPx), catalase (CAT), malondialdehyde (MDA), superoxide dismutase (SOD), cardiac troponin I (cTnI), cardiac troponin T (cTnT) determination kits were obtained from Nanjing Jianchen Bio-engineering CO., Ltd (Nanjing, China). Nuclear factor E2-related factor 2 (Nrf2), NAD(P)H: quinone oxidoreductase 1(NQO1), Heme Oxygenase-1(HO-1), Kelch-like ECH-associated protein-1(keap-1) antibodies were obtained from Abcam Biological Engineering Co., Ltd (Cambridge, UK). 2,2-Diphenyl-1-picrylhydrazyl (DPPH) was obtained from Sigma Chemical Co., Ltd (St. Louis, MO, USA). HPLC-grade acetonitrile was provided by Tedia Co., Ltd (Ohio, USA). All other chemicals and reagents were analytical grade.

### Isolation and purification of polysaccharide from RG

RG (500 g) was extracted twice with 6000 mL boiling distilled water bath (pH 6.8) for 2 h. Afterwards, the extraction solution was concentrated at 65 °C in a rotary evaporator. The concentrated solution was precipitated by adding 95% ethanol to make the ethanol concentration reach 80%, and then incubated overnight at 4 °C. The precipitate was collected after centrifugation, and then the protein in the fraction was removed using Sewage method [23]. The remained fraction was dialyzed in deionized water to remove small molecules. Then the crude RGP were obtained by lyophilizing the solution.

The further separation and purification of crude polysaccharides according to our previous method [24]. The crude RGP (1 g) was applied to a DEAE-52 column (2.6 cm × 60 cm) eluted with stepwise with distilled water, 0.1 M, 0.3 M, and 0.5 M NaCl at 1.0 mL/min. The content of polysaccharide in the eluate was detected by phenol sulfuric acid method. Then the peak tube with highest polysaccharides content was collected, concentrated, dialyzed and lyophilized. The further purified was fractionated by a Sephadex G-100 chromatography column (2.0 cm × 80 cm) and eluted at a flow rate of 0.5 mL/min with distilled water. Appropriate fractions were collected, concentrated, dialyzed and lyophilized to yield RGP1-1. Then RGP1-1 was stored in a bottle desiccator at room temperature for further study.

### Structure characterization of RGP1-1

#### Component analysis

The total polysaccharides and protein content were evaluated based on our previous method [25]. The monosaccharide compositions of RGP1-1 were estimated by pre-column derivatization HPLC method. The polysaccharide sample (5 mg) was dissolved in 2 M trifluoroacetic acid (TFA). And the solution was hydrolyzed at 110 °C for 8 h. Then, the sample was mixed with an equal amount of 0.3 M NaOH solution. The mixed solution was mixed with an equal amount of 1-phenyl-3-methyl-5-pyrazolone (PMP) methanol solution, and then reacting at 70 °C for 100 min. The solution was extracted with an equal volume of chloroform after the reaction. Subsequently, the supernatant was filtered through the microporous membrane (0.45 μM) for HPLC analysis (Agilent technologies 1260) equipped with a C_18_ column (4.6 mm × 250 mm, 5 μm) and a UV detector. HPLC condition: mobile phase: A: acetonitrile, B:0.05 M KH_2_PO_4_, elution procedure: 0–5 min: 17–82% B, 5–10 min: 82–81% B, 10–30 min: 81–80% B, 30–50 min: 80–83% B; flow rate:1.0 mL/min; detecting wavelengths: 250 nm.

#### Assessment of homogeneity and molecular weight

The homogeneity and molecular weight of polysaccharide was examined by high-performance gel permeation chromatography (HPGPC) on a TSK gel-3000XL column (7. 80 mm × 300 mm, column temperature at 35 °C) combined with Agilent 1260 HPLC system (CA, USA) with an Evaporative Light-Scattering Detector (ELSD, detecting temperature:115 °C). Distilled water was taken as the mobile phase at a flow rate of 1.0 mL/min. The molecular weight was calculated by the calibration curve obtained by using T-series standards dextran (180, 2.7 × 10^3^, 5.25 × 10^3^, 9.75 × 10^3^, 1.305 × 10^4^, 3.68 × 10^4^, 6.465 × 10^4^, 1.3535 × 10^5^, 3.006 × 10^5^, 2.00 × 10^6^).

#### Fourier transform infrared spectroscopy (FT-IR) analysis

The IR spectra as KBr pellets of the polysaccharides were recorded in a range of 4000–400 cm^−1^ by a Fourier transform infrared spectrophotometer (Nioletis 5, USA).

#### Methylation analysis

Methylation analysis of polysaccharide was performed in details according to previous method with some modifications [26]. The polysaccharide (5 mg) was dissolved in dimethyl sulfoxide (DMSO, 2 mL) and stirred at room temperature. Afterward, dry NaOH powder (100 mg) was added in it, and the mixture was stirred sequentially for 1 h in anoxic environment. Then, methyl iodide (1 mL) was added dropwise, and the mixture was reacted for 2 h in the darkness by stirring. The mixture was extracted with dichloromethane. Then, the fully methylated polysaccharide was hydrolyzed with 2 M of TFA (2 mL) at 120 °C for 2 h. And the sample was reduced with NaBD_4_and acetylated by acetic anhydride. Then, the partially methylated alditol acetates (PMAA) were analyzed with GC–MS (7890 N/5975B GC–MS, Agilent, USA).

#### Nuclear magnetic resonance (NMR) analysis

Polysaccharide sample was dissolved in D_2_O (99.9%) and deuterium exchange through lyophilization three times for NMR analysis. NMR spectra of polysaccharide (^1^H, ^13^C, COSY, HSQC and HMBC) were conducted on a Bruker 600 MHz NMR spectrometer (Bruker, Rheinsteten, Germany) at 25 °C, respectively.

#### Transmission electron microscope (TEM) and scanning electron microscope (SEM)

The polysaccharide solution in proper concentration was immersed in a water bath at 45 °C for 15 min. And it was dripped immediately on a mesh copper network. A drop of uranyl acetate solution was added to dropwise and dyed for 10 min. The morphology of the polysaccharide was observed by a JEM-2100 TEM (JEOL, Japan). The surface of polysaccharide was performed by SEM (S-3400 N, Hitachi, Japan). Polysaccharide was deposited directly on an aluminum strip and spattered with gold for 1 min. And the sample was analyzed in vacuum with an acceleration voltage.

#### Atomic force microscopy (AFM) analysis

Polysaccharide sample was dissolved in deionized water (20 μg/mL). The sample solution was dispersed on freshly cracked mica and dried at room temperature. The AFM analysis was performed by MFP-3D apparatus (Asylum Research, USA) in tapping-mode.

#### Congo red analysis

The helix–coil transition of polysaccharide was used Congo red test under an alkaline solution. RGP1-1(5 mg) was dissolved with 2 mL deionized water, and then the Congo red solution (80 μM) was added and mixed by a vortex. NaOH solution (1 M) were added to the mixture while the concentration of NaOH was reached to 0–0.5 M. Then the mixture solutions were analyzed with a UV spectrophotometer (Shimadzu Co., Japan) at the range of 400–800 nm.

### Physicochemical properties of polysaccharide

#### Particle size and *zeta* potential analysis

The particle size and *zeta* potential of polysaccharide (5 mg/mL aqueous solution) was analyzed by Zetasizer nano ZS laser particle size analyzer (Malvern, Worcestershire, UK). The dynamic light scattering (DLS) program was used to determine the distribution of average particle size at 632.8 nm. The electrophoretic light scattering (ELS) program was used to determine the *zeta* potential.

#### Thermogravimetric and differential scanning calorimetry (DSC) analysis

The temperature and calorimetry calibration were processed before experiments. Thermo-gravimetric (TG) and DSC analysis of polysaccharide sample (5 mg) was placed in crucible and heated from room temperature to 500 °C at 20 °C/min under a nitrogen (30 mL/min) in a thermogravimetric analyzer (STA200, Netzsch, Germany).

### Analysis of antioxidant activity in vitro

#### DPPH radical scavenging activity assay

The DPPH radical scavenging capacities of polysaccharide was assessed according to the reported methods [27]. In brief, 100 μL polysaccharide solution (0–10 mg/mL) was added into 200 μL DPPH solution, and the mixed samples were incubated in darkness for 30 min. Then, the mixed samples were determined by a microplate reader (Thermo, USA) at the wavelength of 517 nm. Ascorbic acid (Vc) was used as positive control. The DPPH radical-scavenging effect of the sample was calculated by the following formula:

$${\text{DPPH radical - scavenging rate}}\left( \% \right) = \left[ {{{\left( {{\text{A}}_{{\text{c}}} - {\text{A}}_{{\text{s}}} } \right)} \mathord{\left/ {\vphantom {{\left( {{\text{A}}_{{\text{c}}} - {\text{A}}_{{\text{s}}} } \right)} {{\text{A}}_{{\text{c}}} }}} \right. \kern-\nulldelimiterspace} {{\text{A}}_{{\text{c}}} }}} \right] \times 100\%$$

where, A_s_: absorbance of sample, A_c_: absorbance of blank control (ethanol replaces the sample solution).

#### Hydroxyl radical scavenging activity assay

The hydroxyl radical scavenging capacities were determined according to the previous methods [28]. In brief, 1 mL polysaccharide solution (0–10 mg/mL) was mixed with 1 mL ferrous sulfate solution (9 mmol/L), 1 mL salicylic acid solution (9 mmol/L) and 0.5 mL hydrogen peroxide solution (0.1%), then the mixed samples were incubated in 37 °C for 30 min. Subsequently, the samples were determined at the wavelength of 510 nm. Ascorbic acid (Vc)was used as positive control. The hydroxyl radical-scavenging effect of the sample was calculated by the following formula:

$${\text{Hydroxyl radical - scavenging rate}}\left( \% \right) = \left[ {{{\left( {{\text{A}}_{0} - {\text{A}}_{1} } \right)} \mathord{\left/ {\vphantom {{\left( {{\text{A}}_{0} - {\text{A}}_{1} } \right)} {{\text{A}}_{0} }}} \right. \kern-\nulldelimiterspace} {{\text{A}}_{0} }}} \right] \times 100\%$$

where, A_1_: absorbance of sample, A_0_: absorbance of blank control (purified water replaces the sample solution).

### Superoxide ion radical scavenging

1 mL polysaccharide solution (0–10 mg/mL) was mixed with Tris–HCl solution (pH 8.2, 50 mmol/L), purified water and pyrogallol solution (3 mmol/L). Then the mixed solutions were incubated in room temperature for 5 min and 0.5 mL of hydrochloric acid solution (8.0 mmol/L) was added to terminate the reaction. The samples were determined at the wavelength of 325 nm. Ascorbic acid (Vc) was used as positive control. The superoxide ion radical-scavenging effect of the sample was calculated by the following formula:

$${\text{Superoxide ion radical - scavenging rate}}\left( \% \right) = \left[ {{{\left( {{\text{A}}_{0} - {\text{A}}_{1} } \right)} \mathord{\left/ {\vphantom {{\left( {{\text{A}}_{0} - {\text{A}}_{1} } \right)} {{\text{A}}_{0} }}} \right. \kern-\nulldelimiterspace} {{\text{A}}_{0} }}} \right] \times 100\%$$

where, A_1_: absorbance of sample, A_0_: absorbance of blank control (purified water replaces the sample solution).

### Evaluation of protective activity of RGP1-1 for MI injury in vivo

#### Animal model

MI mice model was established according to our previous reports [29]. ICR mice (8–10 weeks, 20 ± 2 g, male) were purchased from Nanjing Qinglongshan animal breeding ground (Nanjing, China). All mice were given distilled water randomly and fed for 3 days at a temperature of 25 °C and relative humidity of 45%. All animal handling procedures were conducted strictly in accordance with the regulations of the P.R. China on the use and care of laboratory animals. All protocols for experiments were approved by the Institutional Animal Care and Ethics Committee of China Pharmaceutical University.

60 mice were randomly divided into 6 groups (*n* = 10/group): group1: blank group, group2: model group (treated with ISO, 60 mg/kg), group3: positive control groups (treated with propranolol, 40 mg/kg), Groups 4, 5, and 6, ISO combined with RGP1-1 (100, 200, and 400 mg/kg, respectively). Mice in group 1–3 were dosed intragastrically with the same volume of distilled water, and group 4–6 were dosed intragastrically with RGP1-1 (100, 200, and 400 mg/kg, respectively) for 14 day. Within 2 h of RGP1-1 administration of on days 12, 13, and 14, mice in groups 2 to 6 were injected with ISO (60 mg/kg), while group 1 was injected with saline. After 24 h fasting, blood samples obtained from the orbital sinus were centrifuged at 3000×*g* at 4 °C for 10 min, and the supernatants were stored at − 70 °C. Then, sacrificing the mice, heart of mice was quickly and meticulously frozen in liquid nitrogen or fixed immediately in a 10% (w/v) formalin solution for further analyses.

### Myocardial infarction size assay

TTC staining was used to assay myocardial infarction size. The specific method is as follows: the heart tissue was evenly cut into 5 pieces in a vertical direction, then placed into TTC solution, and incubated in a constant temperature water bath at 37 °C for 20 min. After that, the tissue was fixed with 4% formaldehyde for 12 h, and photographed and recorded. Normal tissue was stained in red and ischemic tissue in white. Image-J was used for analysis to calculate the myocardial ischemia area.

### Biochemical indicators assay in the serum

The activities of cTnT, cTnI, CK, CK-MB, AST, LDH were assayed in serum using kits following manufacture's protocols. The activity of SOD, GPx, CAT and the content of MDA in serum were assayed using respective kits according to the manufacture's protocols.

### Inflammatory factors assay in the serum

The contents of TNF-α and IL-6 were detected in serum using respective kits according to the manufacture's protocols.

### ROS level assay in the heart tissue

Mice hearts were homogenized (1:10 w/v) in a cold 0.9% NaCl solution. Then, the solution was centrifugated at 3500 rpm for 10 min, and the supernatant was collected for determination of ROS. The ROS level was detected using kits according to the manufacture’s protocols.

### Histopathological examination

HE staining: Briefly, the heart tissues were fixed in neutral paraformaldehyde and sliced into paraffin-embedded sections. And the sections were dewaxed with xylene, different concentration of ethanol, water sequentially, and stained with hematoxylin and eosin (H&E). And then the sections were sealed with neutral resin and observed the morphology under a microscope (Olympus IX71; Olympus Corporation, Japan).

Masson staining: The heart tissues were preserved by fixation with Bouin's solution for 12 h, then dehydrated with a gradient ethanol solution, embedded in paraffin and sliced. Then the sections were dewaxed with xylene and dehydrated with ethanol. Additionally, the sections were placed in a 0.5% iodine solution, and reacted with sodium thiosulfate and Weiger's iron hematoxylin dyeing solution. The sample was stained by ponceau red acid and phosphomolybdic acid solution, followed by counter-staining with aniline blue, and treated with 1% glacial acetic acid. The tissue sections were observed under an inverted microscope.

### Cardiomyocyte apoptosis analysis

The paraffin-embedded heart sections were prepared and stained with the terminal deoxynucleotidyl transferase dUTP nick end labeling (TUNEL) assay kit. Briefly, after dewaxing and rehydration, the heart sections were incubated with proteinase K, terminal deoxynucleotidyl transferase enzyme, anti-digoxigenin, successively. Then, the slices were stained with 4,5-diamino-2-phenylindole (DAPI). Images were randomly observed using a fluorescence microscope (Axio Observer A1, Zeiss, Germany).

### Western blotting

Briefly, cardiac muscle tissues were washed with pre-cooled saline, then the whole protein was extracted on ice. The protein concentration was measured using Bradford method. Equal amounts of protein from each group were denatured and subjected to SDS-PAGE perform protein separation, and transferred to a polyvinylidene fluoride membrane. The membranes were blocked with 5% (w/v) non-fat milk powder at room temperature for 2 h, then incubated with primary antibodies (Nrf2, NOQ1, HO-1, keap1, GAPDH) (diluted in PBS at 1:200) at 4 °C overnight. Subsequently, the membranes were incubated with the secondary antibody at room temperature for 2 h to develop color and then analyzed using ImageJ software (Bio-Rad, USA). The protein levels were expressed as a percentage of the control, and analyzed using Gel-Pro Analyzer software [30]. The relative protein expression was expressed by the ratio of the gray value of the target protein band to the gray value of the internal reference protein band.

### Statistical analysis

All experiments were repeated three times. Data were analyzed using one-way ANOVA by the statistical package (GraphPad PrismTM 5.0) and expressed as mean ± SD. Student's t-test was used for comparison between two groups. *P* < 0.05 was considered to be statistically significant.

## Results and discussion

### Isolation and purification of polysaccharide

**Fig. 1:**
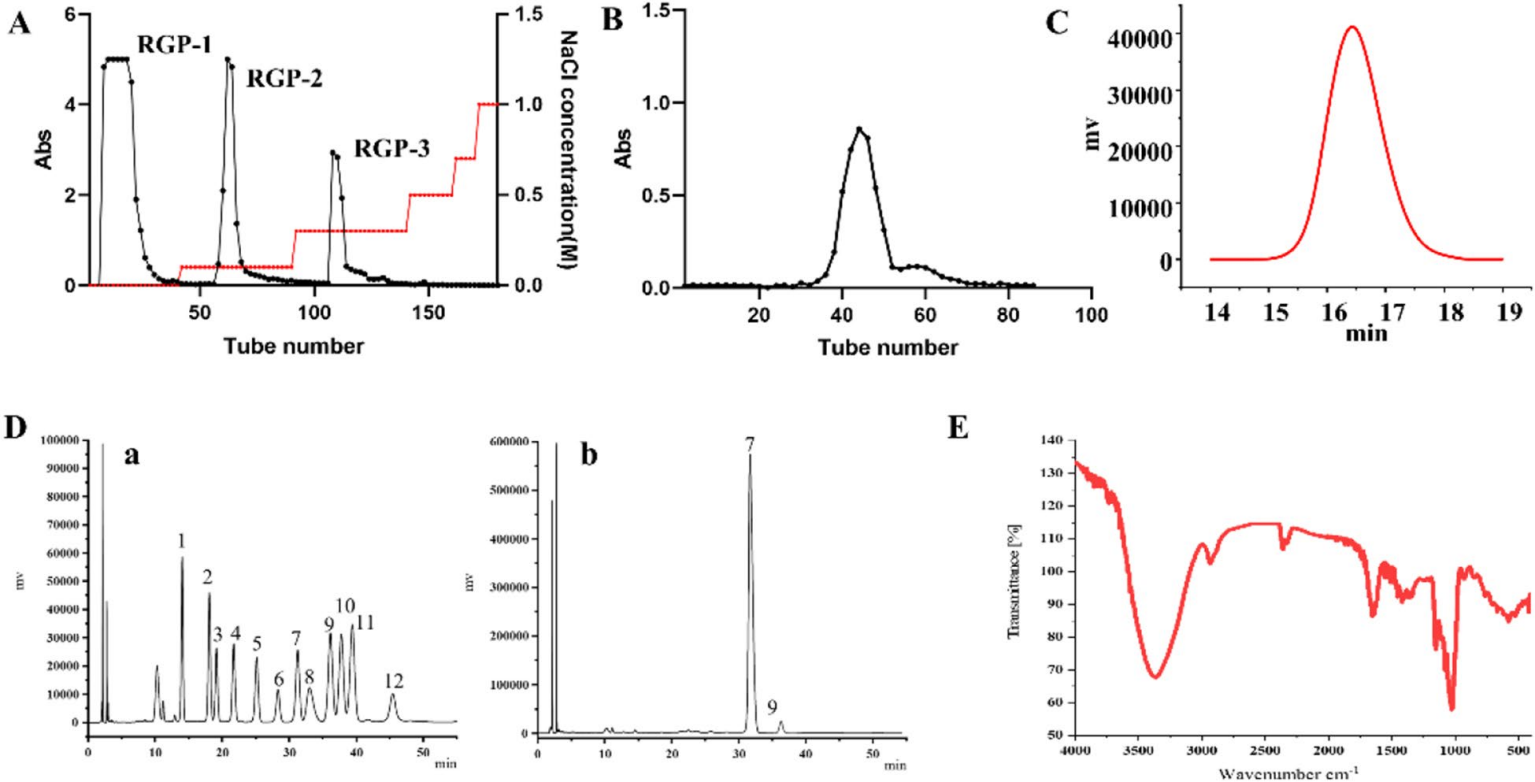
Separation and characterization of RGP1-1. **A** Chromatogram of the crude RGP on DEAE-52 cellulose column; **B** Gel filtration chromatograms of RGP1-1; **C** HPGPC chromatograms of RGP1-1; **D** Chromatograms of monosaccharide compositions (**a** PMP derivative of monosaccharide reference substances; **b** PMP derivated of RGP1-1 sample; 1-Man, 2-Rib, 3-Rha, 4-GlcA, 5-GalA, 6-NAG, 7-Glc, 8-NAGA, 9-Gal, 10-Xyl, 11-Ara, 12-Fuc); **E** Fourier transform infrared spectrum of RGP1-1

The polysaccharide fraction was extracted from RG by water extraction and alcohol precipitation. Then the crude RGP was obtained after removal of proteins and small molecules with a yield of 18.46%. Afterwards, RGP-1 (water fractions) was isolated from RGP by DEAE-52 column (Fig. 1A). RGP-1 was further separated by Sephadex G-100 column (Fig. 1B) and a homogeneous polysaccharide (RGP1-1) was obtained. The carbohydrate content of RGP1-1 was 95.68% according to the determination by the phenol–sulfuric acid assay. The UV scanning spectrum at 200–400 nm showed that the polysaccharide had no absorption at 280 nm and 260 nm, indicating absence of protein.

### Structural characterization of polysaccharide fractions

#### Molecular weight and monosaccharide composition analysis

As shown in Fig. 1C, the chromatogram of RGP1-1 was a symmetrical signal peak which indicating the RGP1-1 was homogeneous, and the molecular weight was 5655 Da according to the calibration curve obtained from a series dextran. As shown in Fig. 1D, standard monosaccharides PMP derivatives was separated within 50 min by HPLC. The monosaccharide composition analysis indicated that RGP1-1 was composed of Glc and Gal in the ratio of 94.26:4.92. Sun et al. reported a red ginseng polysaccharide was composed of Gal, Glc and Ara with the molar ratio of 3:95.3:1.3 [31]. And it indicated the Glc was dominant sectors in RG purified polysaccharides. In addition, the different in composition may be caused by raw materials and purified methods.

#### FT-IR analysis

As shown in Fig. 1E, the infrared spectra obtained a typical and strong wide stretching peak at 3389.43 cm^−1^ for O–H stretching vibration, an absorption peak at 2945.45 cm^−1^ was caused by the telescopic vibration of the C–H stretching vibration of –CH_2_– [32]. The absorption peak at 1417.85 cm^−1^ was caused by the variable-angle vibration peak of C-H [33]. The characteristic absorption band in the 1000–1200 cm^−1^ region might be caused by the stretching vibration of C–O–C of glycosidic structures [34]. The absorption peaks a 1078.36 cm^−1^ indicated a pyranose form of sugar [35]. Furthermore, the peak at 891.74 cm^−1^ was caused by α-d-glycosidic bonds. These results indicated that RGP1-1 was possessed typical absorption peaks of polysaccharides.

#### Glycosidic linkages analysis

The fully methylation product of RGP1-1 was hydrolyzed and acetylated for GC–MS analysis. The peaks of total ions chromatography (TIC) were identified by compared with standard PMAA spectra patterns and other literatures (Additional file 1: Fig. S1). The methylation analysis of each linkage patterns and the molar ratio of sugar residues were shown in Table 1. The methylation results shown that RGP1-1 contained four different glycosidic linkages T-Linked-Glc*p*, 1,4-Linked-Glc*p*, 1,4,6-Linked-Glc*p* and 1,4-Linked-Gal*p* in the approximate ratio of 31.7:31.6:30.5:4.9. The ratio of these residues was accord with monosaccharide composition of RGP1-1. Furthermore, the molar ratio of branching point and terminal units was 1.03. And it was consistent with the fact that the number of terminal units is roughly equal to the number of branching points. Based on above results, the structure of RGP1-1 may be recognized as a branched polysaccharide, and the backbone was mostly composed of → 1)-Glc*p*-(4 → and → 1)-Gal*p*-(4 → repeating units and amounts of → 1)-α-D-Glc*p*-(4 →, T-linked-D-Glc*p* branches.

**Table 1:** The methylation results of RGP1-1

No	t_R_(min)	PMAA	Linkage pattern	Mass fragments (*m/z*)	Molar ratio
1	17.875	2,3,4,6-Me_4_-Glcp	T-Linked-Glcp	217, 205, 162, 145, 129, 118, 102, 87, 71, 59, 43	31.7
2	19.951	2,3,6-Me_3_-Glcp	1,4-Linked-Glcp	277, 233, 173, 162, 129, 118, 113, 102, 87, 71, 59, 43	31.6
3	20.365	2,3- Me_2_-Glcp	1,4,6-Linked-Glcp	233, 189, 173, 162, 129, 118, 113, 102, 99, 87, 71, 59, 43	30.5
4	22.041	2,3,6-Me_3_-Galp	1,4-Linked-Galp	261, 231, 201, 186, 162, 159, 142, 126, 118, 102, 99, 85, 87, 59, 43	4.9

#### NMR analysis

**Fig. 2:**
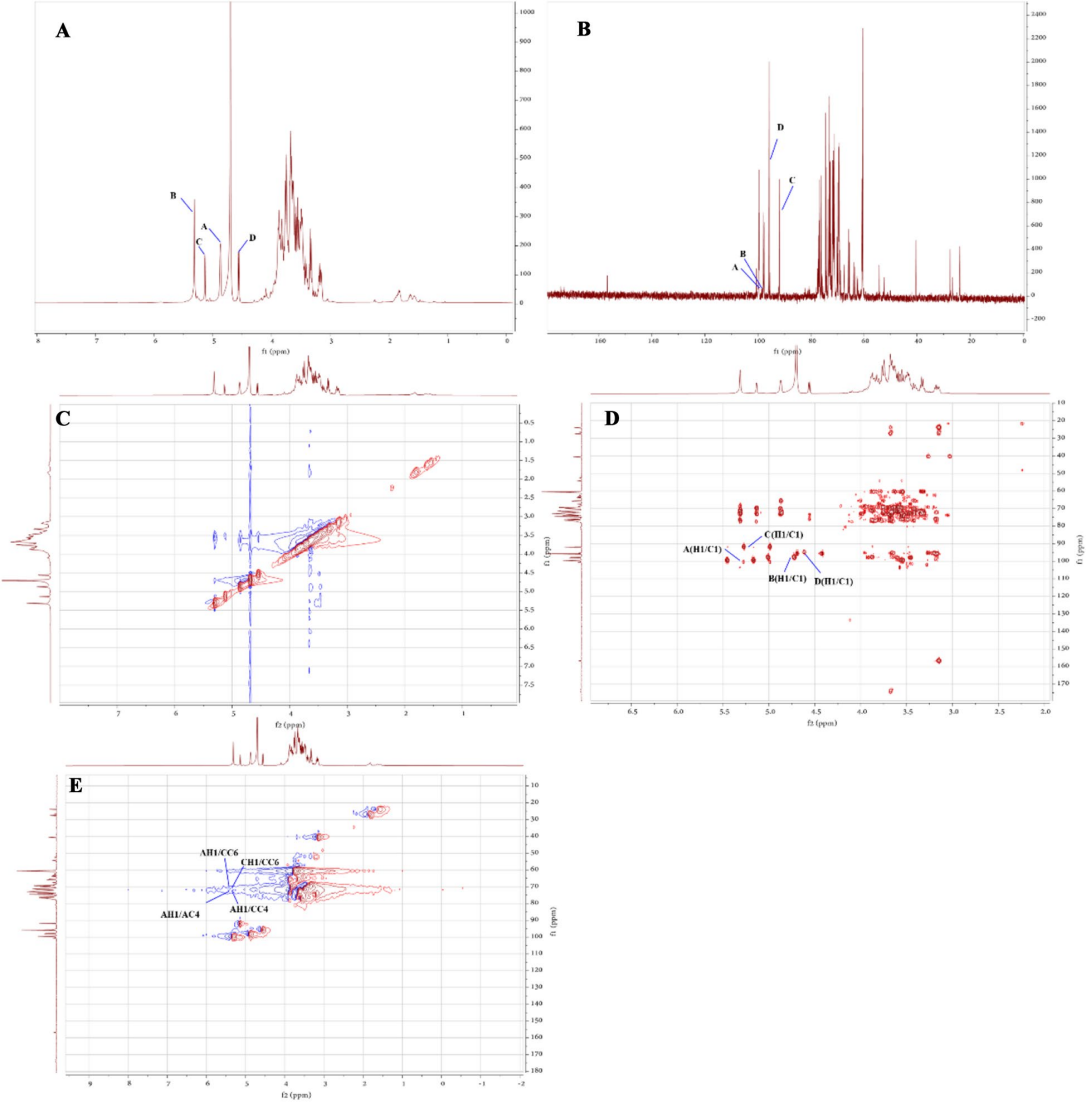
NMR spectra of RGP1-1. **A** ^1^H spectrum; **B** ^13^C spectrum; **C** ^1^H-^1^H COSY spectrum; **D** HSQC spectrum; **E** HMBC spectrum

NMR was applied to characterize the precise structure of RGP1-1 (Fig. 2 and Table 2) according to the NMR spectra and data of previous study [36].

**Table 2:** ^1^H and ^13^C NMR chemical shifts of RGP1-1

Sugar residues		Chemical shifts δ (ppm)					
		1	2	3	4	5	6
*α*-1,4-Glcp (A)	H	5.31	3.51	3.75	3.60	3.79	3.85
	C	99.76	71.28	72.68	72.86	71.46	60.74
*β*-T-Glcp (B)	H	4.87	3.55	3.74	3.57	3.78	3.76
	C	98.7	71.46	72.84	70.20	72.68	60.79
*α*-1,4,6-Glcp (C)	H	5.13	3.59	3.62	3.87	3.57	3.75
	C	91.88	72.83	72.18	76.69	73.64	71.16
*β*-1,4-Galp (D)	H	4.55	3.58	3.66	4.03	3.96	3.71
	C	95.88	71.66	72.68	77.59	71.75	60.91

In the ^1^H NMR spectrum (Fig. 2A), four anomeric protons were distinguished at 5.31, 4.87, 5.13 and 4.55 ppm which were corresponded to H-1of A, B, C and D respectively. The chemical shifts of anomeric protons were attributed to α-pyranose forms. In the ^13^C NMR spectrum (Fig. 2B), the main anomeric carbon signals were at 99.76, 98.70, 91.88 and 95.88 ppm corresponding to C-1 of residue A, B, C and D, which were within the range of 93–105 ppm [37]. It also indicated that four sugar residues were contained in RGP1-1. In the heteronuclear singular quantum correlation (HSQC) spectrum (Fig. 2D), the cross-peaks of 5.31/99.76, 4.87/98.70, 5.13/91.88 and 4.55/95.88 ppm appeared in the anomeric region which were assigned to A-1, B-1, C-1 and D-1 respectively, and the data were also consistent with ^1^H and ^13^C NMR spectra. The other C/H chemical shifts of all monosaccharide residues in RGP1-1were assigned (Table 2) according to the above results, 2D NMR spectra and data of previous study [38–40].

Residue A, B and C was attributed as α-1,4-Glc*p*, β-T-Glc*p* and α-1,4,6-Glc*p* on account of the anomeric shifts. The scales of anomeric C and H combined with the literatures indicated the 5.31/99.76 ppm chemical shift belongs to β-T-Glc*p* residue, the 4.87/98.70 ppm chemical shift belongs to α-1,4-Glcp residue and the 5.13/91.88 ppm chemical shift belongs to α-1,4,6-Glcp residue, and this assignment was also consistent with monosaccharide composition. Compared with previous studies, the other chemical shift signals were obtained from ^1^H-^1^H Cosy, HSQC and heteronuclear multiple bond correlation (HMBC) [41, 42]. The cross peaks of 5.31/3.51(H-1/H-2), 3.51/3.75(H-2/H-3), 3.75/3.60(H-3/H-4), 3.60/3.79(H-4/H-5), 3.79/3.85(H-5/H-6) in β-T-Glc*p* residue, the cross peaks of 4.87/3.55(H-1/H-2), 3.55/3.74(H-2/H-3), 3.74/3.57(H-3/H-4), 3.57/3.78(H-4/H-5), 3.78/3.76(H-5/H-6) in α-1,4-Glc*p* residue, 5.13/3.59(H-1/H-2), 3.59/3.62(H-2/H-3), 3.62/3.87(H-3/H-4), 3.87/3.57(H-4/H-5), 3.57/3.75(H-5/H-6) in α-1,4,6-Glc*p* residue were detected in ^1^H-^1^H homonuclear chemical shift correlation spectroscopy (COSY) spectrum (Fig. 2C). According to the chemical shift of residue H-1 and the HSQC spectrum (Fig. 2D), the chemical shifts of C-2 to C-6 of residue can be obtained, and they were also identified in ^13^C spectrum. The C-1 and C-4 chemical shift of α-1,4-Glc*p* residue were 99.76 ppm and 72.86 ppm respectively, the signals migrated to low-field which proved the possible link sites. The signal shifts of C-4 (δ 76.69 ppm) and C-6 (δ 71.16 ppm) of α-1,4,6-Glc*p* residue migrated to low-field which demonstrated C-4 and C-6 may be the substitute site, and it was consistent with the results of methylation analysis.

Residue D was assigned to be β-1,4-Gal*p* according to the literature [43] and the chemical shifts of anomeric region signal appeared at 4.55/95.88 ppm (H1/C1) (Fig. 2). And it indicated that residue D was in β configuration. The rest carbon and proton signals were distributed as 3.58/71.66, 3.66/72.68, 4.03/77.59, 3.96/71.75 and 3.71/60.91 corresponded to H2/C2, H3/C3, H4/C4, H5/C5 and H6/C6d from HSQC spectra. In addition, down-field signal at C-4 (77.59 ppm) indicated that there was a group attached to the C-4 position of residue D, which was consistent with the methylation results.

The connection sequence between residues can be judged from the HMBC spectrum (Fig. 2F), owing to there are cross peaks between residues and within residues. The anomeric carbon signal at 5.31 ppm of residue A has a correlation peak with its C-4 (72.86 ppm) indicated that the linkage type was presented as → 4)-α-D-Glc*p*-(1 → 4)-α-D-Glc*p*-(1 →. The anomeric hydrogen signal at 5.31 ppm of residue A has a correlation peak with C-6 (71.16 ppm) of residue C, suggesting the presence of → 4)-α-D-Glc*p*-(1 → 6)-α-D-Glc*p*-(1 →. The H-1 (δ 5.31 ppm) of residue A has a correlation peak with C-4 (76.69 ppm) of residue C, suggesting the presence of → 4)-α-D-Glc*p*-(1 → 4)-α-D-Glc*p*-(1 →. The H-1 (5.13 ppm) of residue C also correlates with its own C-6 (71.16 ppm), suggested that they were linked with each other in the form of → 4)-α-D-Glc*p*-(1 → 6)-α-D-Glc*p*-(1 →.

**Fig. 3:**
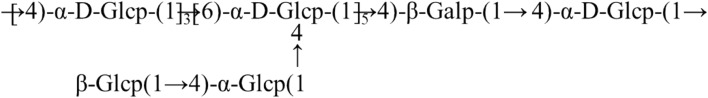
Proposed structure of RGP1-1

Combination of monosaccharide, methylation and NMR analysis in consideration, the possible repeating structure of RGP1-1 was predicted and drawn in Fig. 3. Zhang et al. [44] reported a new homogenous starch-like glucans named WGPN-N 1 which extracted from RG, it was elucidated with a molecular weight of 18 kDa. Moreover, these polysaccharides including RG1-1 were elucidated as starch-like polysaccharide owing to their backbone. The different molecular weight and branches in these polysaccharides lead to different biological activities and properties.

#### SEM and TEM analysis

Compared with nucleotides and proteins, polysaccharide had a more complex three-dimensional morphology. Morphology was studied by TEM and SEM.

**Fig. 4:**
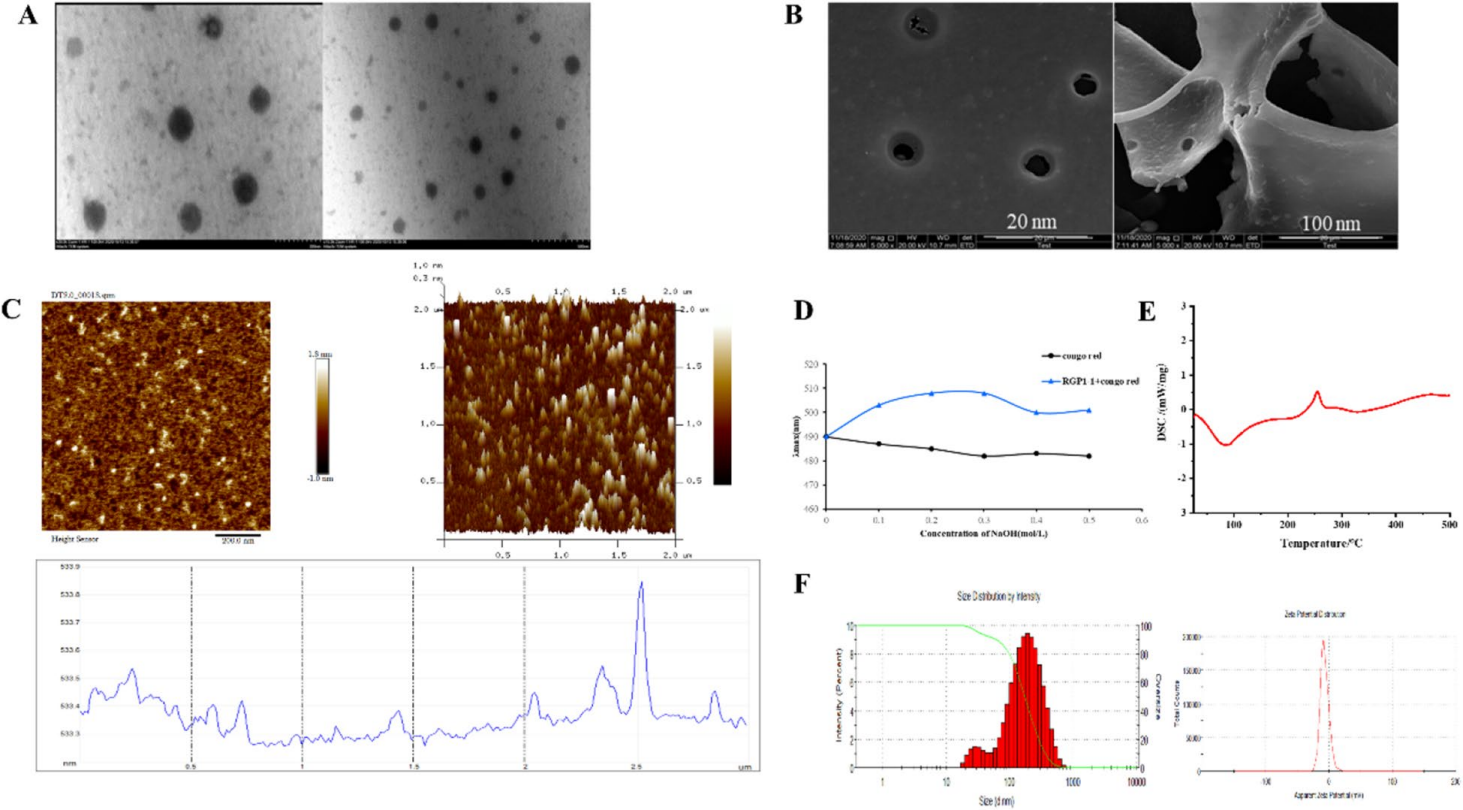
Stereo-shapes and physiochemical properties of RGP1-1. **A** Transmission electron micrographs of RGP1-1; **B** Scanning electron micrographs of RGP1-1; **C** Atomic force micrographs of RGP1-1; **D** Congo red analysis of RGP1-1; **E** The curves of DSC of RGP1-1; **F** Particle size distribution and zeta potential of the RGP1-1 aggregates in water at 1.0 mg/mL

As shown in Fig. 4A, the surface of RGP1-1 has an amorphous morphological and irregular sheet structure under the 500× lens, and relatively smooth and flat under the 2500× lens in SEM. Moreover, the surface of RGP1-1 was smooth and tight along with little holes, and it may reveal powerful intermolecular forces in RGP1-1. In addition, the larger space in the flake layer may provide cohesive space for water molecules which bring on a better solubility, contributing to an increase of its activity [45]. TEM (Fig. 4B) showed that RGP1-1 was spherical structure with a particles size of are 100–200 nm. Drugs of this size may be easily to absorb [46].

#### AFM analysis

AFM was widely used to study the properties and spatial conformation of polysaccharide. In this experiment, AFM was used to examine the morphology of RGP1-1. As shown in Fig. 4C, the 2D and 3D image of RGP1-1 performed an irregular entanglement structure with a height ranged from 0.5–2 nm. In addition, the average height of chains of RGP1-1 were large than 1.0 nm, which was consistent with the Congo red analysis and the triple helix reported previously [47]. Li et al. conducted AFM analysis on the obtained polysaccharides and found that the polysaccharide chains were entangled with each other to form aggregates of various sizes [48]. These entangled appearances in polymers may be caused by van der Waals forces, intramolecular and intermolecular hydrogen bonds.

#### Congo red analysis

Polysaccharides with helical conformation can form complexes with Congo red, and the maximum absorption performed red shift. As shown in Fig. 4D, compared with control group, the maximum absorption of RGP1-1-Congo red complex was increased observably. Interestingly, at the NaOH concentration of 0.35 M, the maximum absorption was decreased which declared the despiralization of RGP1-1 in strong basicity condition. These results suggested that RGP1-1 had triple-helical structure. Moreover, these findings were constituted with other polysaccharide which extracted from *Inonotus obliquus* with triple-helical conformations [49].

### Physicochemical property analysis

#### Thermal properties analysis

Thermostability is an important factor for bioactive molecules and analyzed by thermogravimetric analyzer. As shown in Fig. 4E, the first stage was mainly related to the loss of combined water, which covered from 25 to 200 °C, the mass was reduced by nearly 15.45%. The second stage were chemical reactions and atomic recombination which was performed from 320 to 500 °C. The mass reduced approximatively 75.2%. The DSC was used to detect the thermal changes with the increased temperature. As showed in Fig. 4E, three endothermal peaks were 92.0 °C, 215.6 °C and 336.6 °C, respectively. And the enthalpy changes of system were 12.66 J/g, 4.12 J/g and 9.69 J/g correspondingly. It revealed that RGP1-1 may be structurally stable and had good thermal stability.

#### Particle size and zeta potential analysis

The *zeta* potential is the direction of surface charge and the performance of stability of system. The larger absolute value of *zeta* potential perform that the mixed system is more stable. As shown in Fig. 4F, the *zeta* potential of RGP1-1 solution was 6.82 mV and the average particle distribution of RGP1-1 was 117.4 nm. It revealed the aggregation of polysaccharide molecules and confirmed the AFM analysis. The anionic charge character in polysaccharides may be the cause of this polymerization phenomenon [49].

### Antioxidant activities of polysaccharide fractions in vitro

**Fig. 5:**
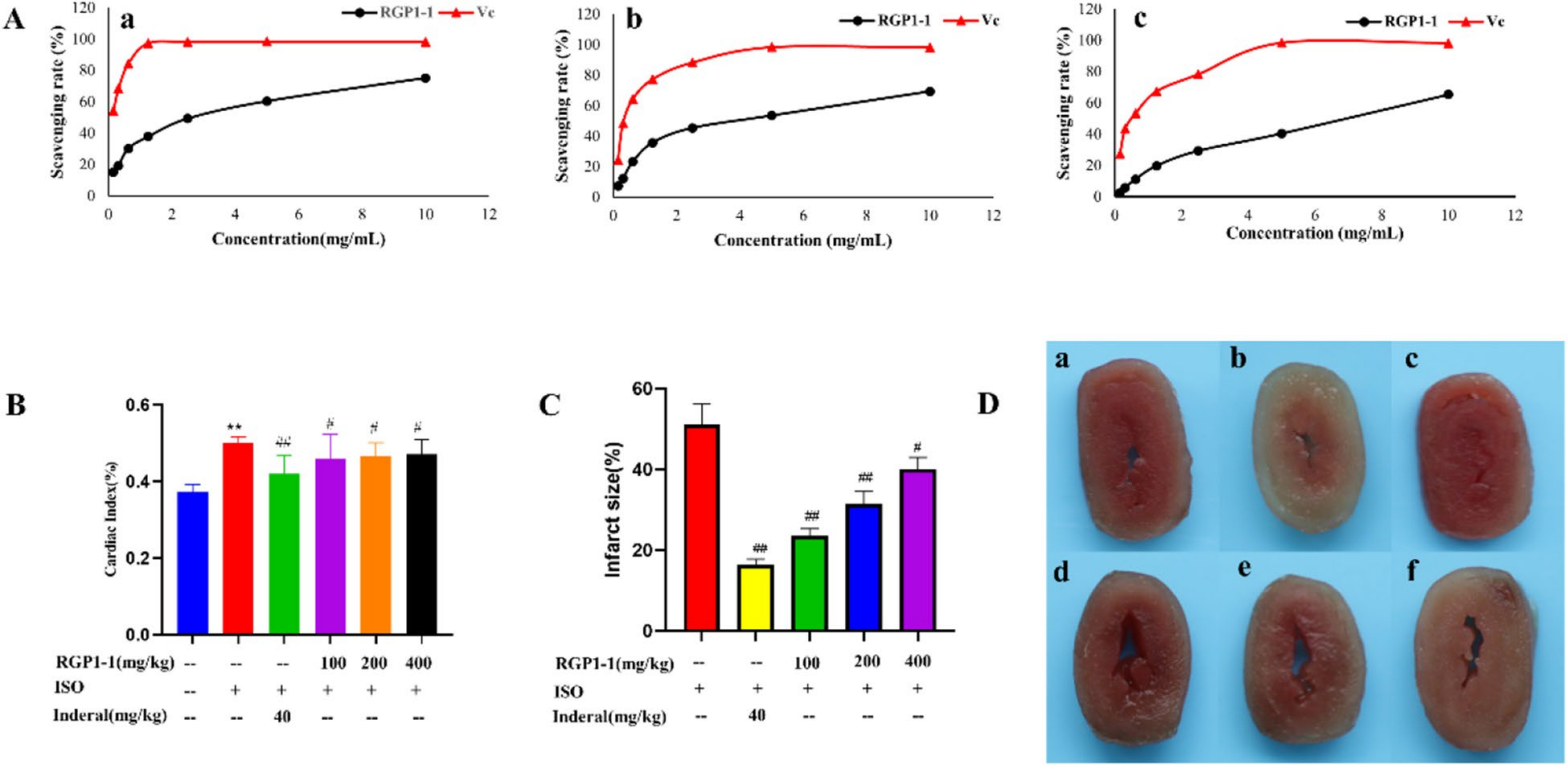
Effect of RGP1-1 on antioxidant activity in vitro, and the area of myocardial infarction in mice. **A** In vitro antioxidant activity assay of RGP1-1 (a-DPPH radical scavenging assay, b-Hydroxyl radical scavenging ability assay, c-Superoxide anion-scavenging activity assay, Values are means ± SD, n = 3); **B** Effect of RGP1-1 on cardiac index of mice; **C**, **D** Effect of RGP1-1 on area of myocardial infarction in mice. (**a** control blank; **b** model group (ISO); **c** inderal group; **d** low-dose RGP1-1; **e** medium-dose RGP1-1; **f** high-dose RGP1-1). The data are presented as means ± SD (n = 10 mice per group). *vs*. blank control, ^##^*P* < 0.01, ^##^*P* < 0.05, *vs*. model control, **P < 0.01, *P < 0.05

DPPH, hydroxyl radical and superoxide anion assay were stable and widely used methods to estimate the free radical scavenging ability of polysaccharide. RGP was proved to have good antioxidant activity [8]. In order to estimate the antioxidant activities of RGP1-1, DPPH, hydroxyl radical and superoxide anion free radical scavenging experiments were applied for study. As shown in Fig. 5A, with the increase of RGP1-1 concentration, the scavenging free radical rate also increases gradually. It indicated the antioxidant activity of RGP1-1 was in dose-dependent manner within the range of 0.125–10 mg/mL. The highest scavenging rate of DPPH, hydroxyl radical and superoxide anion were at the concentration of 10 mg/mL, and it was 75.33%, 69.33% and 65.36%, respectively. In brief, RGP1-1 has a strong ability to scavenge free radicals. In addition, it can provide bonding electrons, and resulting in radical scavenging activity [50]. Besides, the mechanism of hydroxyl radical scavenging of polysaccharide was responsible for the interaction of hydrogen with radicals, and followed by a termination of the radical chain reaction. Unfortunately, the details of this mechanism have not yet been elucidated [51].

### Polysaccharide fractions protect against ISO-induced MI injury in mice

#### Effects of polysaccharide fractions on heart index

Isoproterenol was a β-adrenergic agonist which can induce myocardial ischemia through increasing the force and frequency of myocardial contractions [52]. In this study, an animal model induced by ISO to estimate the protective effect of RGP1-1 on MI. As shown in Fig. 5B, the heart index of model group was higher than the blank group, indicating increase in size and generate oedema of heart. Compared with model group, the heart index of mice pre-treated with RGP1-1(100, 200 or 400 mg/kg), showing a significant reduction (*P* < 0.01). In the Inderal pre-treated group (positive group), the heart index had significantly decreased, when compared with model group (*P* < 0.01). It was stated that the heart weight increment of mice induced by ISO may according to the increased water content, edema intermuscular space and connected with onset of myocardial necrosis [53]. The pretreatment of RGP1-1 obviously decreased the heart weight in ISO-induced mice, and it revealed RGP1-1 had protective effect on ISO-induced MI in mice.

#### Effects of polysaccharide fractions on area of myocardial infarction

The myocardial TCC results of the mice were shown in Fig. 5C, D. The myocardial tissue of the blank group was ruddy, and there was no obvious area of white infarction. Compared with blank group, the myocardial tissue in the model group had large area of myocardial infarction which the was 51.02 ± 5.15% (*P* < 0.01). After the pre-treated with Inderal (positive group), the infarct area was reduced to 16.41 ± 1.37% (*P* < 0.01). In addition, the myocardial infarction area of RGP1-1 groups (100 mg/kg, 200 mg/kg, 400 mg/kg) were significantly reduced to 23.54 ± 1.78%, 31.54 ± 3.03%, 40.09 ± 2.91%, respectively. These results indicated that polysaccharide fraction can significantly improve the blood condition during the course of onset of myocardial ischemia, reduce myocardial tissue infarction, and exert its protective effect on myocardial ischemia injury.

#### Effects of polysaccharide fractions on serum marker enzymes

Heart is one of the most active organs which contains a mass of cardiac troponin and enzymes. cTnT and cTnI were regulatory protein of myocardial muscle tissue contraction and a marker of myocardial injury and necrosis. Its elevated level indicates that myocardial tissue and function were damaged. It can be used for the diagnosis and evaluation of myocardial ischemia. Moreover, cytosolic enzymes such as AST, LDH, CK, and CK-MB were usually use as diagnostic markers of myocardial tissue injury. These cellular enzymes in serum during the occurrence of myocardial ischemic reperfusion can reflect severity of cardiomyopathy and loss of functional integrity and permeability of cell membrane [54, 55]. The activities of serum biomarkers were characterized to evaluate the cardioprotective effect of RGP1-1 in ISO-induced mice.

**Fig. 6:**
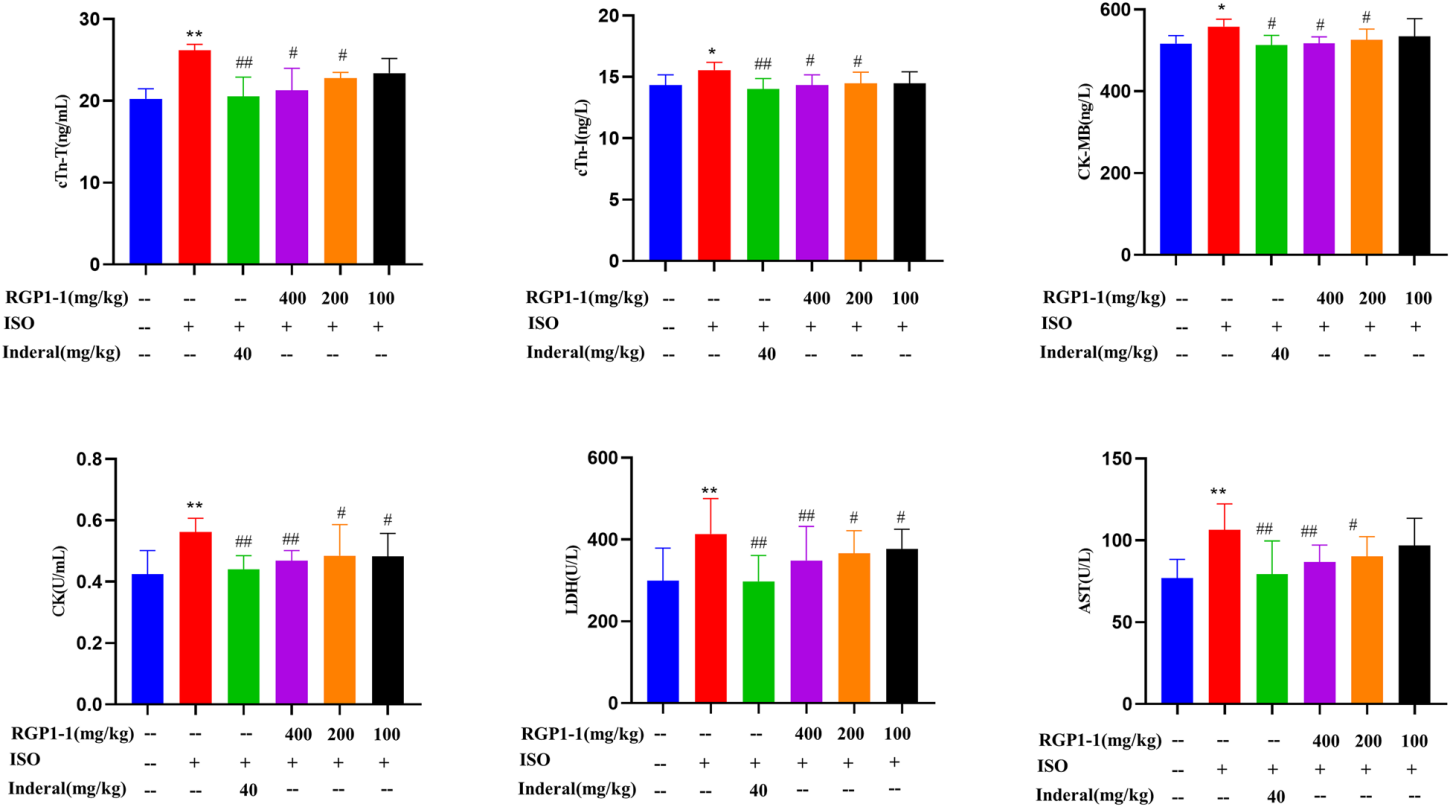
Effect of RGP1-1 on the cardiac enzyme activity. The data are presented as means ± SD (n = 10 mice per group). *vs*. blank control, ^##^*P* < 0.01, ^##^*P* < 0.05, *vs*. model control, **P < 0.01, *P < 0.05

As shown in Fig. 6, compared with blank group, the cardiac troponin (cTnT, cTnI) in model group were significantly increased (*P* < 0.01), it indicated myocardial tissue were damaged. Interestingly, while pre-treated with Inderal or polysaccharide fraction, these cardiac troponins showed an obvious reduction in activities compared with model group. Besides, As shown in Fig. 6, the marker enzymes (AST, LDH, CK and CK-MB) in the serum of mice in model group were significantly increased (*P* < 0.01). And the increased activities of these enzymes in serum owing to their leaked from the heart as a result of myocardial necrosis induced by ISO. When pre-treated with Inderal or RGP1-1(100, 200 and 400 mg/kg, respectively), these enzymes in serum showed an obvious reduction in activities when compared with model group. These results indicated that RGP1-1 could maintain cellular membrane integrity and permeability. Consequently, it could reduce the leakage of these enzyme and attenuate ISO-induced tissue injury.

#### Effects of polysaccharide fractions on serum antioxidant activity

**Fig. 7:**
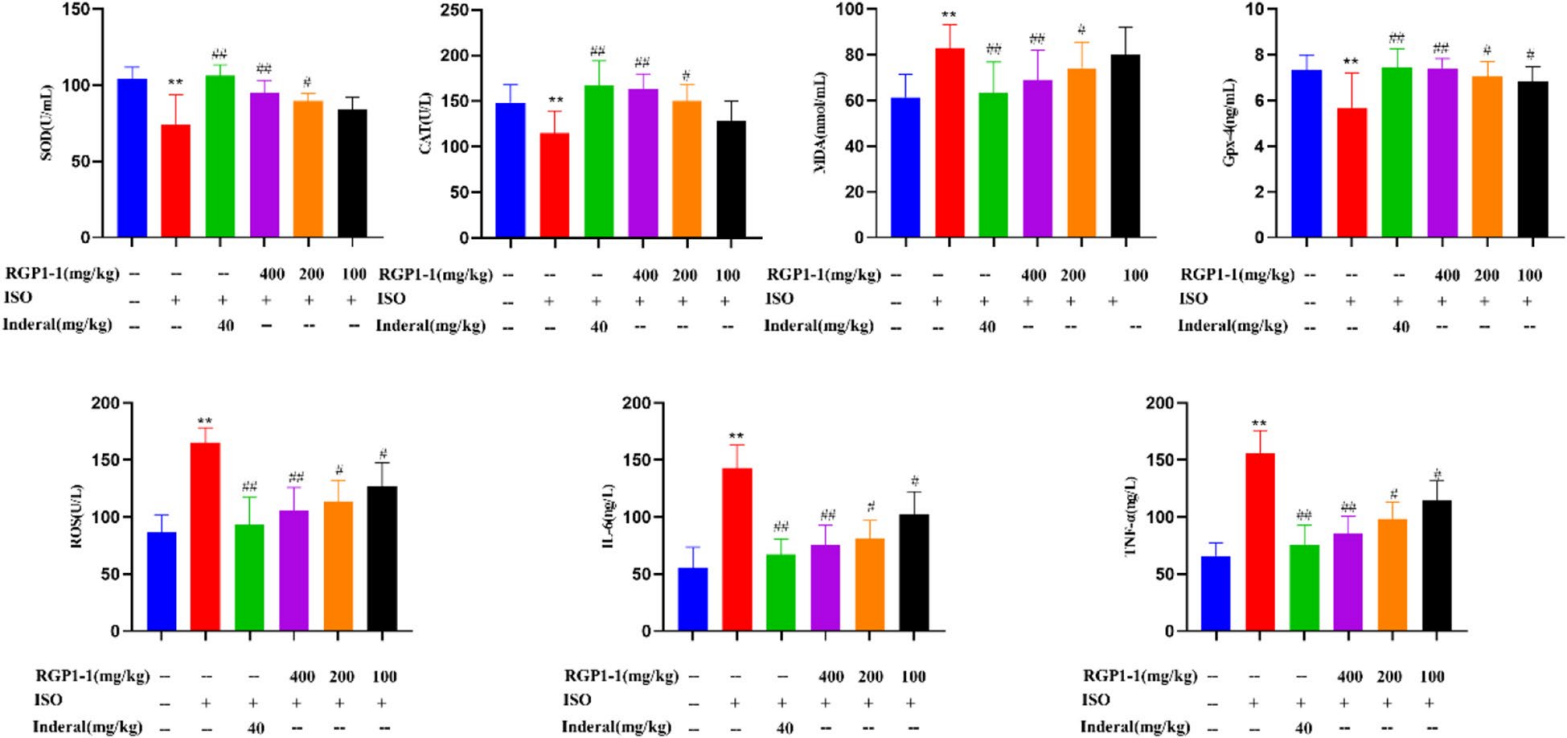
Effect of RGP1-1 on oxidative stress biochemical markers and inflammatory factors. The data are presented as means ± SD (n = 10 mice per group). *vs*. blank control, ^##^*P* < 0.01, ^##^*P* < 0.05, *vs*. model control, **P < 0.01, *P < 0.05

Heart damage was related to the oxygen free radicals which could damage various biological targets [56]. In order to illuminate the protective effect of RGP1-1 on MI injury, antioxidant activity of RGP1-1 was evaluated. As shown in Fig. 7, compared with blank group, the activities of SOD, CAT and GPx in serum of mice in model group were observably decreased, along with an increase of MDA (*P* < 0.01). On the contrary, compared with model group, the activities of SOD, CAT and GPx in serum of mice in RGP1-1 administration group showed a significant increase (*P* < 0.05 or *P* < 0.01), while a significant decrease in MDA (*P* < 0.05 or *P* < 0.01). These results proved for the first time that administration of RGP1-1 could effectively attenuate anti-oxidant stress damage in ISO-induced mice.

The role of oxidative stress in the pathophysiological mechanism of ischemic heart disease and ISO-induced MI injury has been fully demonstrated [57]. In the antioxidant defense system, free radical scavenging enzymes such as SOD, GPx and CAT are the first line of defense. Some eliminating reactive oxygen radicals such as superoxide anion, hydrogen peroxide, and hydroxyl radical can protect the tissue from oxidative damage [58]. A significant marker of oxidative stress is reduction in the SOD level, and SOD was usually existed in the plasma membrane. CAT was used as a catalyst for the removal of hydrogen peroxide [59]. And GPx protects the cellular membranes from the peroxidative damage by reducing hydrogen peroxide and lipid peroxides [60]. Inhibiting these enzymes leads to the accumulation of these oxidants, making the myocardial cell membrane more susceptible to oxidative damage. Pretreatment with RGP1-1 obviously increase the activities of these enzymes in serum, which suggest the antioxidant activity of RGP1-1 in ISO-induced mice.

MDA was regarded as an index of cellular damage and cytotoxicity [55]. The levels of MDA in serum of mice in model group was significantly increased when compared to blank group. While pretreatment with RGP1-1, the MDA content was decreased in serum when compared to model group. These results might be owing to the enhanced activities in antioxidant enzymes (SOD, GPx and CAT), and the free radicals were effectively scavenged.

The above findings indicated that RGP1-1 could greatly improve cellular anti-oxidative stress effect. And this result due to the therapeutic action of RGP1-1 in against peroxidative injury. These results suggested that RGP1-1 provided important protection against ISO-induced MI in mice by enhancing endogenous antioxidant activity.

#### Effect of polysaccharide fractions on ROS level

In the process of MI, the mitochondrial electron transport chain was destructed and led to generation of a large amount of reactive oxygen species (ROS). And the antioxidant enzymes such as SOD and CAT that use ROS as substrates were over-consumed. As a result, ROS cannot be reduced and metabolized which produced accumulation. ROS could attack nucleic acids, proteins and other macromolecules, destroy biofilm unsaturated fatty acids, and generate biotoxic MDA [61]. Inhibition of ROS production which is an effective mean to improve oxidative stress damage in MI.

In this study, the anti-oxidative stress activity of RGP1-1 polysaccharide was evaluated by estimated the inhibitory effect of ROS. As shown in the Fig. 7, ROS level in model group was significantly enhanced. After giving RGP1-1, the level of ROS was significantly down-regulated which indicated the production of ROS was inhibited, it suggested that RGP1-1 had the effect of protecting oxidative damage.

#### Effect of polysaccharide fractions on inflammatory factor content

After MI, a large number of pathologically stimulated inflammatory factors are released. Among them, TNF-α and IL-6 are used as inflammatory chemokines, which can stimulate inflammation and damage myocardial tissue. IL-6 could activate inflammatory cells, aggravate the inflammatory response, and stimulate vascular endothelial cells to release ROS [62]. TNF-α is a pro-inflammatory cytokine, which can induce the release of other inflammatory mediators, and can significantly promote apoptosis [63]. In this study, the anti-inflammatory effect of RGP1-1 polysaccharide was evaluated by estimated the content of TNF-α and IL-6 in serum. As shown in Fig. 7, compared with blank group, the content of TNF-α and IL-6 in serum of mice in model group were observably increased (*P* < 0.01). On the contrary, compared with model group, the content of TNF-α and IL-6 in serum of mice in RGP1-1 administration group showed a significant decrease (*P* < 0.05 or *P* < 0.01). These results proved that administration of RGP1-1 has an antagonistic effect on inflammation, and may indirectly inhibit oxidative damage and resist apoptosis.

#### Effects of polysaccharide fractions on cardiomyocyte apoptosis

**Fig. 8:**
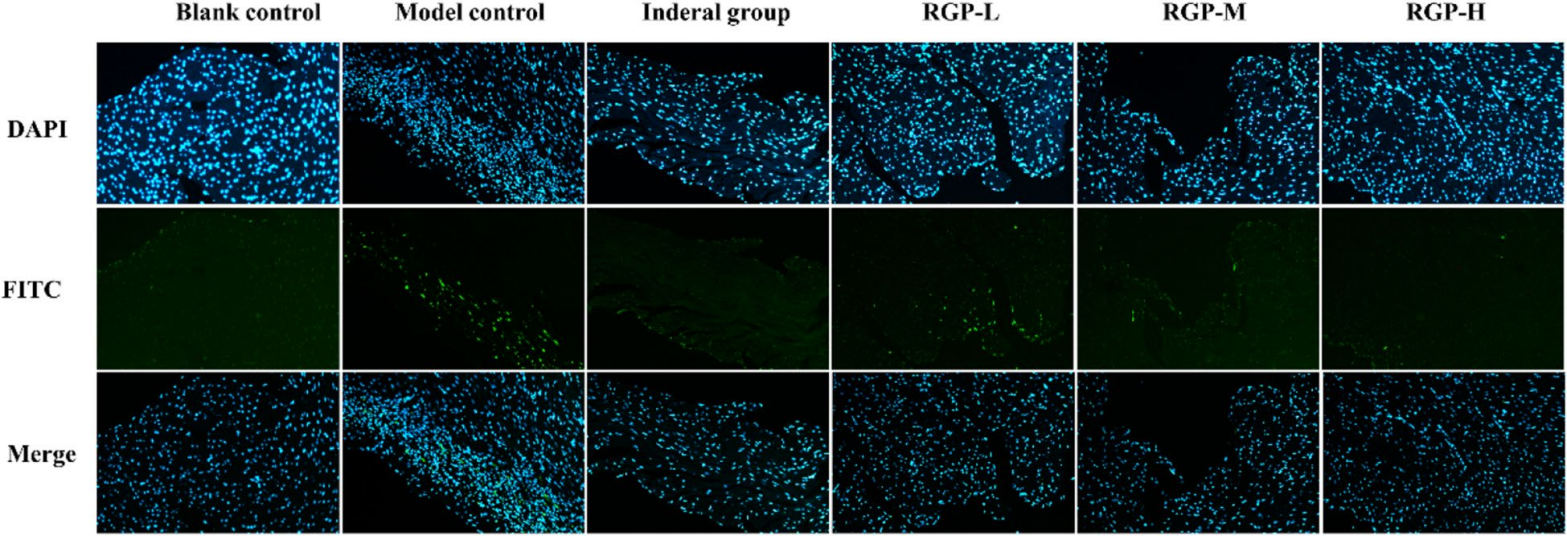
Effect of RGP1-1 on apoptosis of cardiomyocytes. The data are presented as means ± SD (n = 10 mice per group). *vs*. blank control, ^##^*P* < 0.01, ^##^*P* < 0.05, *vs*. model control, **P < 0.01, *P < 0.05

The TUNEL assay was applied to evaluate the effect of RGP1-1 on cardiomyocyte apoptosis. ISO caused DNA fragmentation in nucleosomes. As shown in Fig. 8, compared with blank group, a number of TUNEL-positive cells increased obviously in model group. And it indicated many cardiomyocytes produced apoptosis in the model group. The results of RGP1-1 pretreatment group (100, 200 and 400 mg/kg, respectively) showed that the number of TUNEL-positive cells significantly reduced when compared with model group. Positive group (Inderal) pre-treated also showed the similar results with RGP1-1. These results suggested that RGP1-1 could inhibit ISO-induced apoptosis by the mitochondria-dependent apoptotic pathway.

#### Effects of polysaccharide fractions on myocardial histopathology

**Fig. 9:**
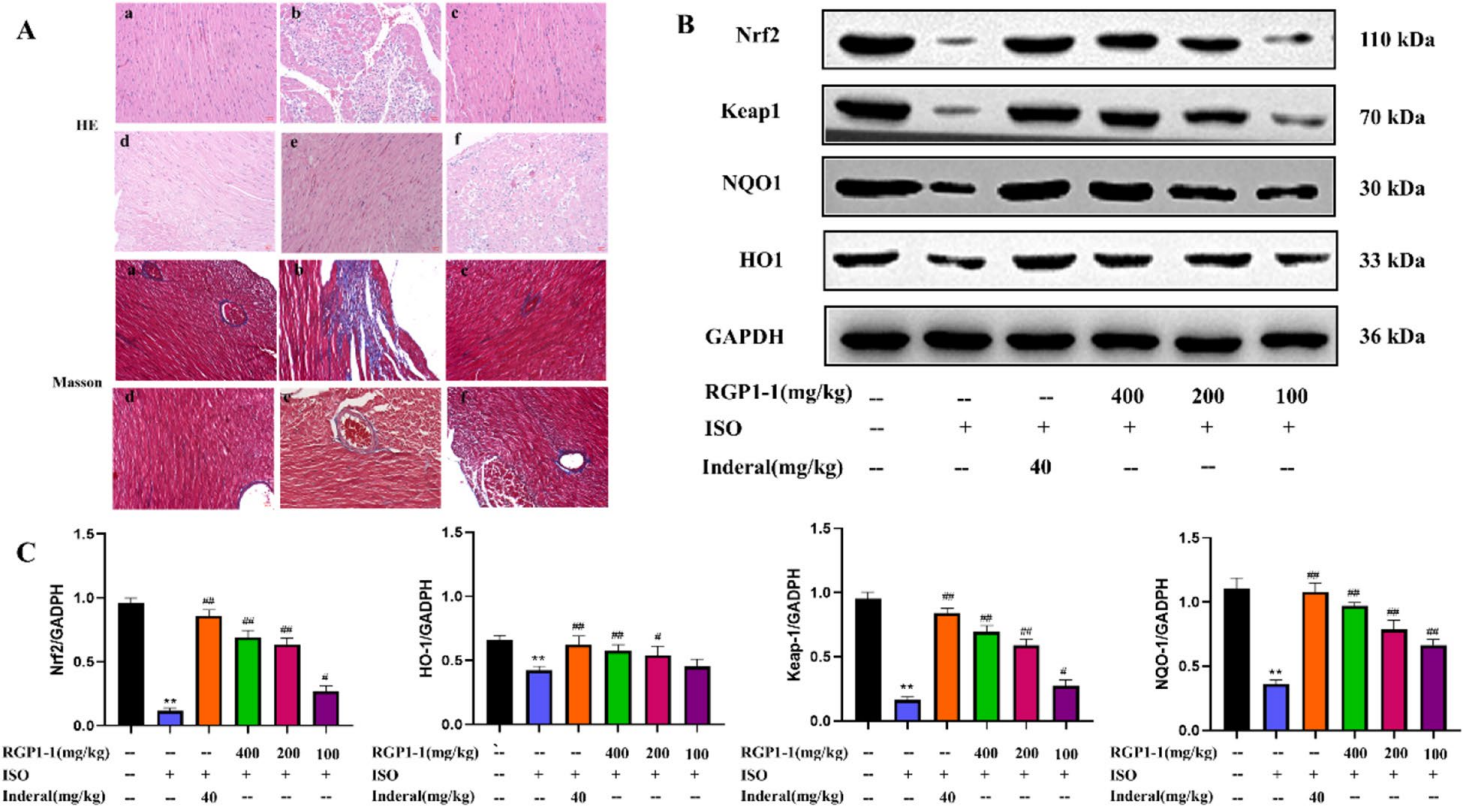
Effect of RGP1-1 on morphological changes and western blot analysis for examining the expressions of Nrf2, HO-1, NOQ-1, Keap-1 in cardiac tissues. **A** Representative images of pathological structure of heart in mice (**a** control blank; **b** model group (ISO); **c** inderal group; **d** low-dose RGP1-1; **e** medium-dose RGP1-1; **f** high-dose RGP1-1;HE staining ×400, Masson staining ×400); **B**, **C** Western blot assessments of Nrf2, HO-1, NOQ-1, Keap-1 expression in cardiac tissues. The data are presented as means ± SD (*n* = 10 mice per group). *vs*. blank control, ^##^*P* < 0.01, ^##^*P* < 0.05, *vs*. model control, ^**^P < 0.01, *P < 0.05

Histopathology of heart was examined to evaluated the protective effect of RGP1-1 on the myocardial tissues. As shown in Fig. 9A, compared with blank group, the model group showed histopathological changes in myocardial tissue in HE stained. Specifically, obvious myocardial cell degeneration and necrosis leading to impairment of membrane structural and functional integrity, more inflammatory cell infiltrated. Pretreatment with RGP1-1 (100, 200 and 400 mg/kg, respectively), heart histopathological changes were partially repaired, which indicated that RGP1-1 has significant cardioprotective effects. Photomicrograph of positive group (Inderal) pre-treated also showed the similar results of myocardial tissue with less cellular infiltration and necrosis. Compared with the blank group, model group showed histopathological changes in myocardial tissue with Masson stained. The cells were moderate fibrous tissue hyperplasia and moderate fibrosis, and a large number of inflammatory cells and moderate degeneration and necrosis. The positive group (Inderal) showed a small amount of blue reaction in cardiomyocytes and myocardial interstitial blood vessels, indicating no obvious fibrosis, proliferation, inflammation and degeneration. Cardiac histopathological changes were repaired by RGP1-1 pretreatment (100, 200 and 400 mg/kg, respectively). In addition, there was only a small amount of blue reaction at high doses. It suggested that high dose of RGP1-1 can significantly protect myocardial tissue.

### Polysaccharide fractions regulated Nrf2/HO-1 expression to prevent myocardial membrane injury in mice

The Nrf2 pathway is the most important endogenous anti-oxidative stress pathway which was discovered in recent years. The major transcription factor of expression of antioxidative enzyme genes which was closely related to the improvement of MI injury [64–66]. Generally, there are two ways to exert its antioxidant effect. One is that it can promote the transcription of Nrf2 mRNA and increase the protein synthesis of Nrf2 under oxidative stress. For another, when body was stimulated with some electrophilic substances, ROS or upstream pathways, Nrf2 will dissociate from its chaperone protein Keap1 and activate Nrf2. And the transfer of Nrf2 from the cytoplasm to the nucleus was increased, then bind to the antioxidant response element as a heterodimer with tenofibromin. Finally, it reduced the damage which caused by oxidative oxidative stress by up-regulating the expression of the downstream phase II detoxification enzymes nuclear antioxidant enzyme genes NQO1 and HO-1.

In this study, the level of Nrf2 in the heart tissue was determined by western blot, and the mechanism of RGP1-1 regulating myocardial membrane was explored. As shown in Fig. 9B, C, compared to the blank group, the expression of Nrf2 protein in model group decreased significantly (*P* < 0.01). On the contrary, the results of RGP1-1 pretreatment group (100, 200 and 400 mg/kg, respectively) showed that the expression of Nfr2 protein had been significantly increased (*P* < 0.05) when compared with model group. The result of positive group was similar to the RGP1-1 group. These results suggested that RGP1-1 could prevent myocardial tissue oxidative stress damage in mice, which might be achieved by improvement of Nrf2 protein expression.

The effects of RGP1-1 on improving myocardial injury were evaluated with the disease development-related factors HO-1, NQO1 and keap-1 as the indicators. As shown in Fig. 9B, C, the expression of HO-1, NQO1 and keap-1 protein of the model group decreased significantly which compared to the blank control group (*P* < 0.01). However, positive drug can significantly increase the expression levels of these factors. The increase could also be increased with RGP1-1 pre-treatment. Compared with model group, high dose of RGP1-1 can significantly up-regulate HO-1, NQO1 and keap-1protein expression (*P* < 0.01). The results revealed that RGP1-1 in preventing and treating MI may be through improving antioxidant capacity to protect oxidative damage and regulating the pathway of Nrf2/HO-1.

## Conclusion

A purified polysaccharide RGP1-1 of 5655 Da was successfully extracted, purified and identified from RG, and its physicochemical property and myocardial ischemic injury protection activity were also investigated in this study. RGP1-1 was composed of Glc and Gal in the ratio of 94.26:4.92. The methylation and NMR analysis indicated the backbone chain was → 1)-Glc*p*-(4 → and → 1)-Gal*p*-(4 →, branched partially at O-4 with α-D-Glc*p-*(1 → residue. RGP1-1 performed a triple-helical conformation, flaky and irregular spherical structure with molecule aggregations and stable thermal properties. Moreover, it contained 6.82 mV *zeta* potential, 117.4 nm partical size and polymerization phenomenon. Pharmacological results showed that the effect of RGP1-1 in preventing and treating myocardial ischemia may be through improving antioxidant capacity to protect oxidative damage and regulating the pathway of Nrf2/HO-1. Results suggested that RGP1-1 had a significant effect on the protective effect on myocardial ischemia injure and provided some scientific evidence for the development of this natural resource which could be used as a functional food and a potential alternative medicine.

## Supplementary Information

**Additional file 1: Figure S1.** GC–MS spectra of methylation of RGP 1-1.

## Data Availability

The data set supporting the results of this article are included within the article and Additional files.

## Abbreviations

MI: Myocardial ischemia
RG: Red ginseng
RGP: Red ginseng polysaccharide
TCM: Traditional Chinese Medicine
FT-IR: Fourier transform infrared
DSC: Differential scanning calorimetry
TEM: Transmission electron microscopy
SEM: Scanning electron microscopy
NMR: Nuclear magnetic resonance
