# Supplementary figures and images for: Characterization of a novel polysaccharide from red ginseng and its ameliorative effect on oxidative stress injury in myocardial ischemia
Source: Chin Med. 2022 Sep 24;17:111. doi: 10.1186/s13020-022-00669-6 (PMC9509600; doi:10.1186/s13020-022-00669-6)

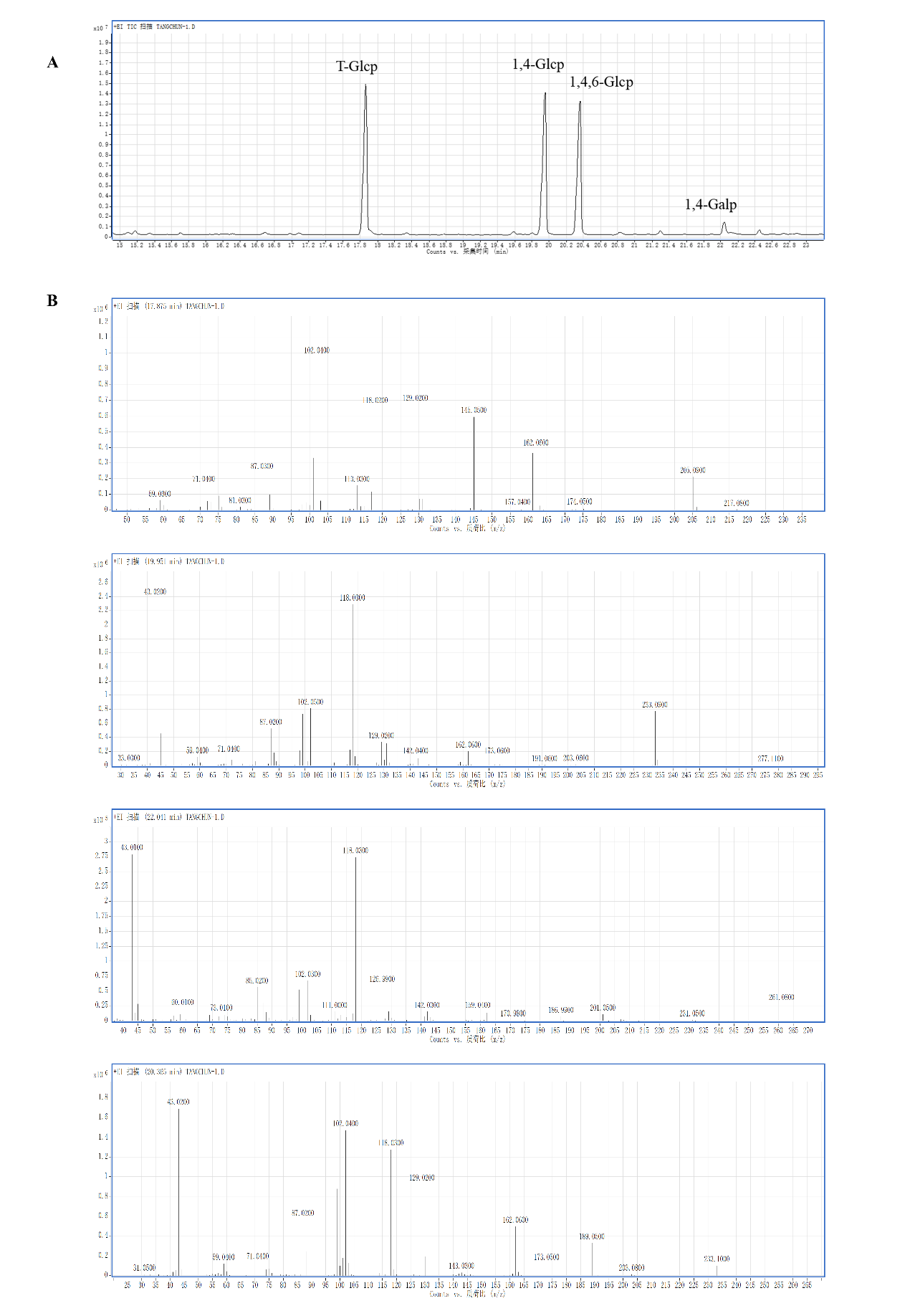


**Figure S1. GC-MS spectra of methylation of RGP 1-1**

Supplement: Supplementary file 1 — Additional file 1: Figure S1. GC–MS spectra of methylation of RGP 1-1. [file 13020_2022_669_MOESM1_ESM.docx]
